# Gambling consumption and harm: a systematic review of the evidence

**DOI:** 10.1080/16066359.2023.2238608

**Published:** 2023-08-02

**Authors:** Viktorija Kesaite, Heather Wardle, Ingeborg Rossow

**Affiliations:** a School of Social & Political Sciences, University of Glasgow, Glasgow, Scotland; b Norwegian Institute of Public Health, Oslo, Norway

**Keywords:** Total consumption model, gambling consumption, risk of harm, risk curves, concentration, gambling expenditure, prevention

## Abstract

The total consumption model (TCM) postulates a close link between total consumption and levels of harm within the population, which has important implications for prevention. This review aimed to explore evidence relating to the application of the TCM and theoretical elements associated with it (i.e. the distribution of harms; the concentration of consumption) to gambling by reviewing evidence pertaining to the distribution of harms across the population; the concentration of gambling consumption; and evidence of the validity of the TCM in gambling. Systematic literature searches were performed using MEDLINE, PsycINFO, and Web of Science databases, restricted to publications between January 1, 2010, and March 29, 2023. The search identified seven studies examining risk curves for gambling harm, of which only two employed continuous consumption measures. This nascent literature suggests mixed evidence for the relationship between gambling consumption (e.g. losses, frequency, expenditure, and expenditure as a percentage of income) and risk of harm. Five publications found that the concentration of gambling consumption was high among those experiencing gambling harms, with some evidence that spending is more concentrated for certain games (e.g. online casinos) than for others (e.g. lotteries). Finally, four studies assessed the TCM, suggesting close association between gambling consumption and problem gambling, lending empirical support to the validity of the TCM. However, robust evidence is nascent and further research is required to assess these relationships.

## Introduction

Globally, commercial gambling opportunities have increased significantly in the last decade. Current estimates suggest that by 2025, annual Gross Gambling Yield globally (the money retained by industry after winnings are paid) will be approximately $550 billion, increasing from around $450 billion in 2016 (GBGC, 2022)^1^. This expansion is occurring due to a dual process. First, many jurisdictions are legislating to permit gambling thus normalizing gambling activity across populations. Second, technology has created new ways to gamble, providing access to commercial gambling products across a variety of online platforms (Parke and Parke 2019).

With increased gambling provision and consumption comes greater concerns about its impact on the health and wellbeing of communities. Gambling is associated with a range of adverse consequences, affecting not only the person who gambles but also their families and communities (Langham et al. 2015). These consequences exist on a spectrum ranging from (but not limited to), financial difficulties to bankruptcy, relationship breakdown, experience of adverse physical and mental health symptoms and suicidality (Langham et al. 2015). The consequences of gambling can be long lasting, affecting people long after gambling has ceased.

Increased recognition of these harms has led to greater demands for more effective policies to prevent them (Johnstone and Regan 2020). Policy makers, regulators and academics alike have looked to other public health areas to assess the extent to which prevention policies from these areas could be applied to gambling. One consideration is the extent to which the Total Consumption Model (TCM), as applied to alcohol and other public health areas, is applicable to gambling (Rossow 2019).

The TCM is conceptualized in two ways (Table 1). The first application, as proposed by Ledermann (1956), predicts a close association between total consumption (e.g. mean per capita alcohol consumption) and excessive levels of consumption (e.g. alcohol consumption above a fixed value). Recent studies have found some evidence to support this in relation to salt intake, blood pressure, alcohol consumption, body mass index (Rose and Day 1990), and in gambling (Rossow 2019).

**Table 1.** Summary of the total consumption model.

	Basic TCM	Extended TCM
Model predictions	Association between total consumption and excessive level of consumption	Association between total consumption and consumption related harm
Examples of the TCM in the previous literature	Blood pressure and hypertension Body mass index and obesity Alcohol consumption and excessive alcohol consumption (Rose and Day 1990; Rossow and Mäkelä 2021) Gambling frequency and excessive gambling frequency (Rossow 2019)	Alcohol consumption and: liver cirrhosis violence suicide (Norström and Ramstedt 2006) Gambling frequency and problem gambling (Rossow 2019)

The second application of the TCM, describes the link between total consumption and consumption-related harms in a given population (Sulkunen et al. 2019). This posits that the total consumption (of some good) in a population is positively associated with the rate of harm from consumption. In other words, when total consumption (or mean consumption) increases, the rate of harm increases as well. Equally, it posits that when total consumption decreases, the rate of harms also decrease. This suggests that the association between total consumption and harm rate might be mediated by the prevalence of heavy consumers, who are most at risk of experiencing harms. Thus, the TCM implies that measures that effectively reduce total consumption, will also curb harm rates at the population level.

To further understand the applicability of the TCM model to gambling, it is important to explicate the theoretical elements associated with it. Firstly, it is assumed that across populations or within a population over time the underlying distribution curve for gambling consumption is of a fairly similar shape; it is monotonic and with a long tail on the right side. Thus, when total consumption increases, the tail to the right will be longer, and hence a larger fraction of consumers will exceed a level of excessive consumption. There is strong evidence that this is the case for alcohol consumption (Kehoe et al. 2012). It is also important to understand how skewed the distribution may be. The more skewed the distribution, the larger the fraction of total consumption accounted for by the heaviest consumers. Thus, for a very skewed distribution, variation in the proportion of heavy consumers is bound to covary with total consumption. Whereas for a slightly skewed distribution, this prediction is not as strong. Thus when looking to assess the extent to which the TCM can and should be applied to gambling, it is of interest to consider the extent to which distribution of gambling consumption is skewed.

Secondly, we need to consider how harms are distributed across the population of gamblers. In short, is it only those with excessive consumption who experience harms or are harms incurred by people at lower levels of consumption? This question has implications for prevention policy. Harms that only occur due to excessive consumption support targeted focus on these individuals, who would be viewed as being distinct from others. Harms distributed among people at all levels of consumption, however, suggest policies should target the whole spectrum of consumers. Thus, while the TCM may be valid irrespective of whether harms occur only at very high consumption levels or also at lower levels, its implications for prevention depends on the shape of the risk curves for harms. For example, linear risk curves would suggest that even small amounts of gambling activity increase risk of gambling harms; replicating findings amassed during the last 50 years on the relationship between smoking, and the risk of health consequences and social harm (Maani et al. 2022). Exponential risk curves would imply that individuals with excessive consumption account for most harms. Other types of risk curves include r-shaped, which suggests that risk of harm increases at low levels of consumption and then decreases, or stabilizes after a certain point (Abbott 2020).

To date there has been relatively little systematic evaluation of these elements and of the broader applicability of the TCM to gambling. A prior review by Rossow (2019) examined studies that reported associations between continuous measures of gambling consumption (e.g. mean household gambling expenditure) and either excessive scores on those same continuous gambling consumption measures (e.g. gambling expenditures of more than 10% of household income), or measures of problem gambling identified *via* screening instruments (e.g. Problem Gambling Severity Index scores). Rossow concluded that the included studies supported the TCM and that population-level interventions should be applied to tackle gambling harm, although she expressed caution about the generalizability of these results, as the evidence in this area remains scarce. Several limitations of Rossow’s (2019) review should be noted. It was not carried out according to the Preferred Reporting Items for Systematic Reviews and Meta-Analyses (PRISMA) guidelines. Moreover, while the review provides some insights on the aggregate relationships of the TCM, it did not consider the probability of harm at different levels of consumption at the individual level and the concentration of consumption.

As such, better understanding of how total consumption and gambling harms are related is essential. Our study thus aimed to undertake a systematic review of recent evidence to a) identify and synthesize evidence on the shape and distribution of risk curves between gambling consumption and gambling harm, b) examine the concentration of gambling consumption among the population of gambling consumers, and c) update Rossow’s prior review on evidence applying the TCM to gambling.

Because gambling encompasses a range of commercial products and practices, a secondary objective was to consider whether the concentration of gambling consumption or shape of the risk curves between consumption and harm varies by product. Understanding this would allow us to generate preliminary insights into whether the TCM may be more, or less, applicable to different types of gambling activity.

## Methods

### Search strategy

This review was conducted and reported according to PRISMA (Preferred Reporting Items for Systematic Reviews and Meta-Analyses) guidelines (Moher et al. 2009). The review protocol was not pre-registered but is available in Appendix A of this review. The search strategy included terms on the shape of the risk curves in gambling, the consumption of gambling and its concentration that affect the skewness of the distribution, and the two versions of the TCM (see Appendix A). A summary of the PICO (population, intervention, comparison, outcome) criteria is presented in Table 2. We searched the following databases: Medline, PsycINFO, and Web of Science for articles published between 1st January 2010 and 29th March 2023. We limited papers to those published from 2010 onwards, after the development of the online gambling market to ensure that our review reflects more recent trends in this rapidly changing landscape. All searches were limited to English language articles. To identify any relevant articles in the grey literature, we searched Google Scholar; GambleAware Info Hub; Gambling Commission; GambLib (Gambling Research Library); and other national and international organization websites (see Appendix A). Searching and identification of the relevant studies was supplemented using expert advice (HW, IR).

**Table 2.** A summary of population, Exposures, comparisons, outcomes, and study design criteria.

Criteria	Definition
Population	Children, adolescents, and adults
Exposures	Gambling consumption (e.g. expenditure, frequency)
Comparisons	Gambling modalities; gambling products; age groups; gender; regional disparities
Outcomes	Excessive or heavy gambling measured as a proportion above a certain cutoff point on the same continuous gambling consumption measure Gambling harm measures based on standardized measures
Study design	Longitudinal studies; cross sectional studies with 3 or more sub-samples

The database search results were exported to, and de-duplicated using EndNote (X9) and manually screened by title and abstract by one reviewer (VK), according to a pre-defined inclusion and exclusion criteria presented below (for more detail also see Appendix A). A second validation screening of titles and abstracts was performed by HW. Full texts were retrieved for eligibility and screened by two reviewers (VK; HW). Forward and backward searching of bibliographies/citations of included studies were undertaken.

### Inclusion and exclusion criteria

Studies were included if they were: (i) primary or secondary data analyses on the relationship between gambling consumption (including measures such as gambling frequency; gambling expenditure; and gambling losses) and prevalence of excessive gambling (proportion above a fixed cutoff on a continuous gambling indicator; basic TCM) or gambling harms (self-reported harms based on a validated screening instrument measure; extended version of the TCM); (ii) studies that either used risk curves, concentration indices, or the basic/extended version of the TCM to explain the relationships between total gambling consumption and gambling harm; and (iii) English language papers, reviews, commentaries, and empirical studies published from 2010 onwards. There were no restrictions applied to population nor country. Alongside longitudinal studies, we also included reviews and meta-analyses that provide some insight into the relationships of the TCM. Cross-sectional studies were also included if they provided analyses of at least three population samples, which has been the conventional way of testing for the TCM hypothesis in this literature (see Rossow 2019).

### Data extraction

Data extraction from the included studies was carried out by one reviewer (VK) and a second independent assessment of the included studies was carried out by a second reviewer (HW). Disagreements between reviewers on data extraction were discussed and consensus reached.

Studies meeting the inclusion criteria by full text were classified by theoretical framework, namely the basic and extended versions of TCM, risk curves, and concentration indices. Extracted data included: author, year of publication, country and region of study, dataset, period covered, sample size, population, measure of gambling consumption, measure of excessive gambling, gambling activity, analysis type, methodology, findings (e.g. the shape of the risk curves), and limitations.

## Results

### Characteristics of included studies

Overall, 17 studies were included in this review (see Appendix B). All were based on high-income populations. Three studies each reported data from Australia, the United Kingdom, and Finland respectively. There were two studies each from Canada, and two from mixed national online platforms. There was one study each from Iceland and Norway. Two studies examined data from multiple countries, of which one was a review study which included multiple high-income countries. Most primary studies used cross-sectional datasets (*n* = 13), three carried out analysis using longitudinal datasets and one study was a review which included studies that used both types of datasets. For the majority of analyses, gambling consumption was measured at the same time as gambling harms (*n* = 16) and there was one study that used a lagged measure of gambling consumption. Sample sizes ranged from 809 to 139,152 observations, with the majority of studies analyzing adult age populations (*n* = 15).

**Figure 1.**
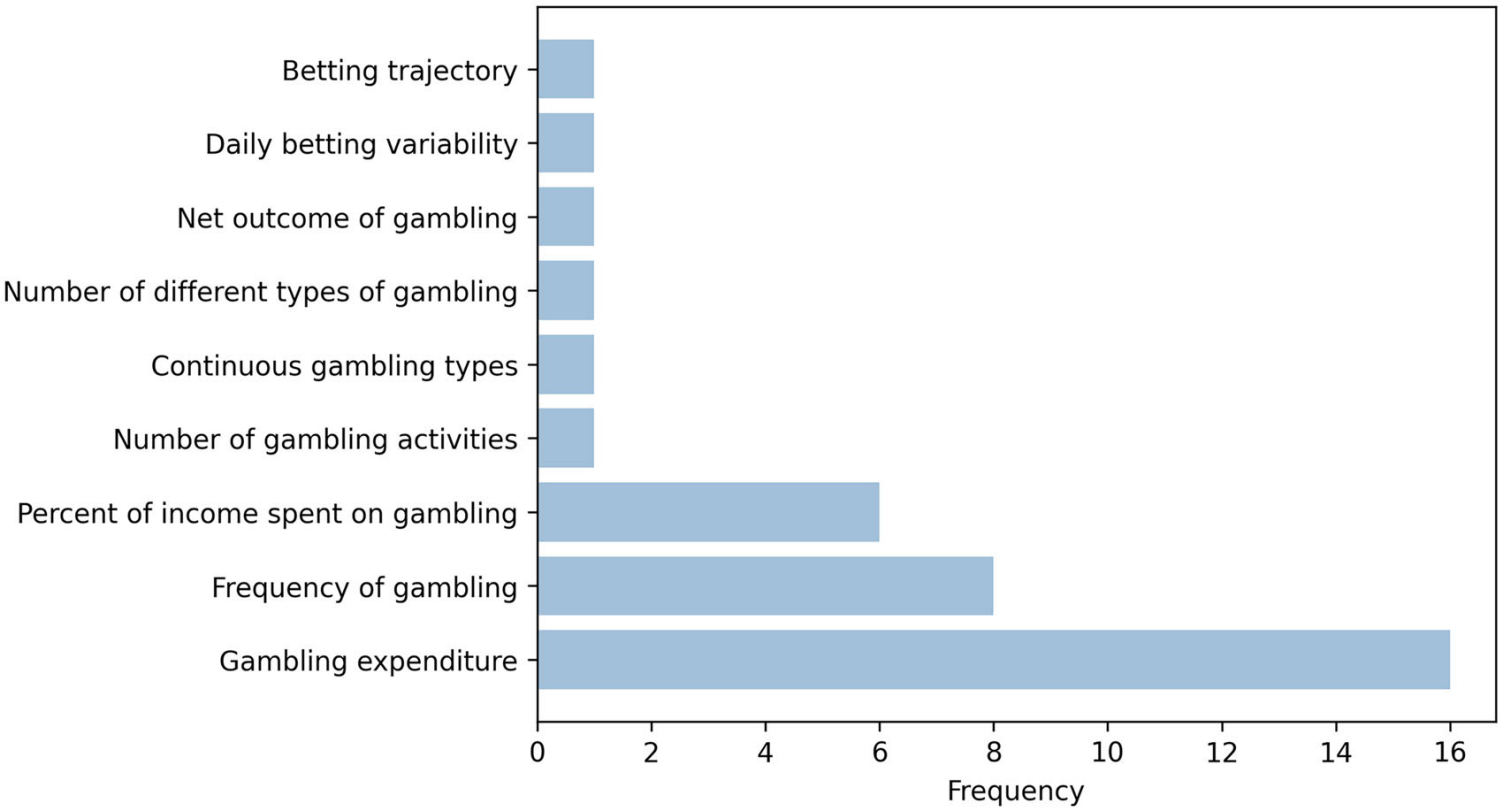
Summary of gambling consumption measures in the included studies^2^.

**Figure 2.**
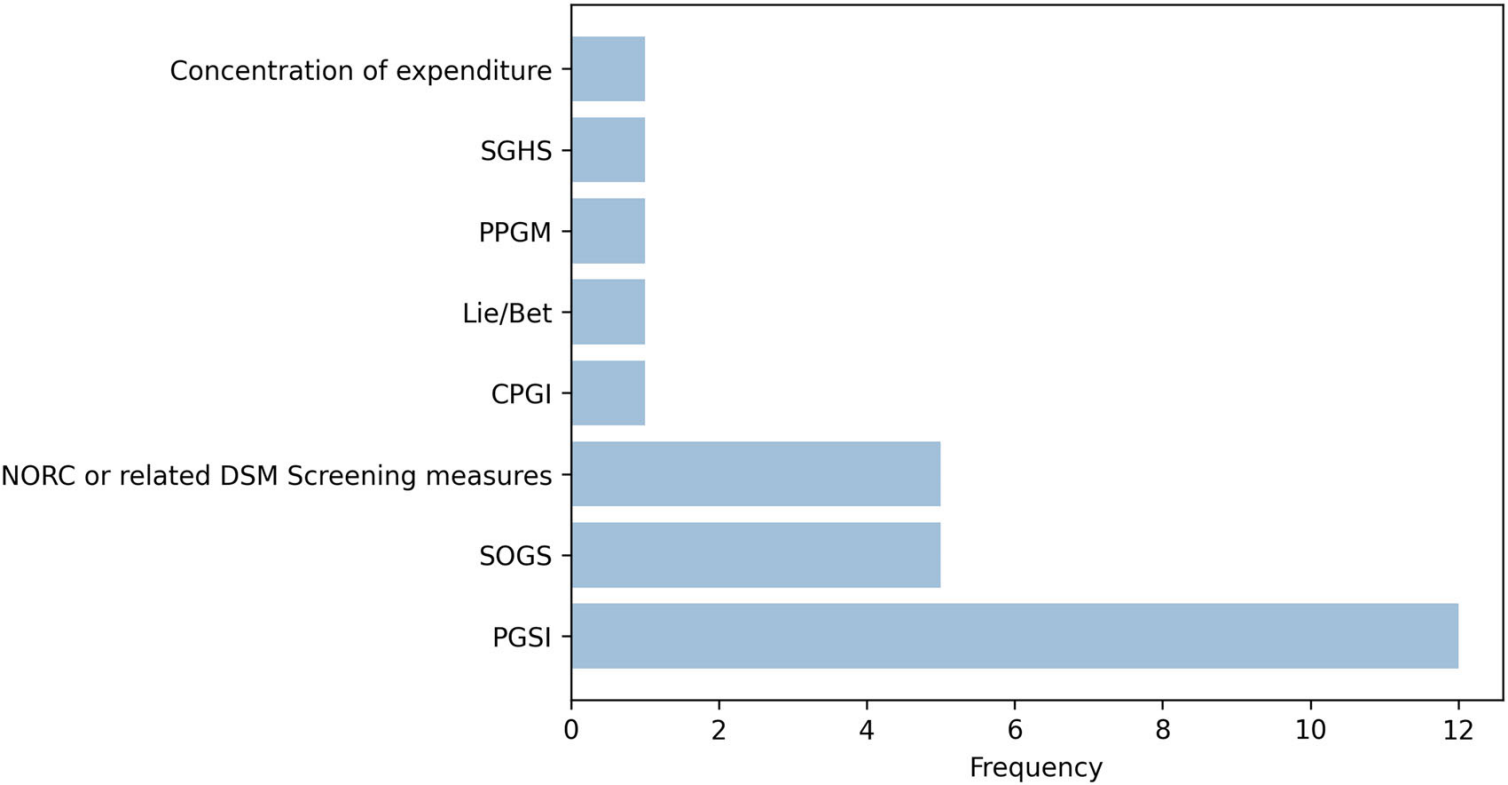
Summary of gambling harm measures in the included studies^3^.

Studies used a variety of gambling consumption measures: gambling expenditure (*n* = 16), frequency of gambling (*n* = 8), percent of income spent on gambling (*n* = 6), and a number of other gambling consumption measures (see Figure 1). For the gambling harm measures, the most frequently used was the Problem Gambling Severity Index (PGSI) (*n* = 12), followed by South Oaks Gambling Screen (SOGS) (*n* = 5), NORC or related DSM Screen for Gambling Problems including the Brief Biosocial Gambling Screen (BBGS) (*n* = 5) and ‘vital few’ concentration of expenditure (*n* = 1). A summary of the exact measures of gambling harms used is presented in Figure 2.

### Risk curves

**Figure 3.**
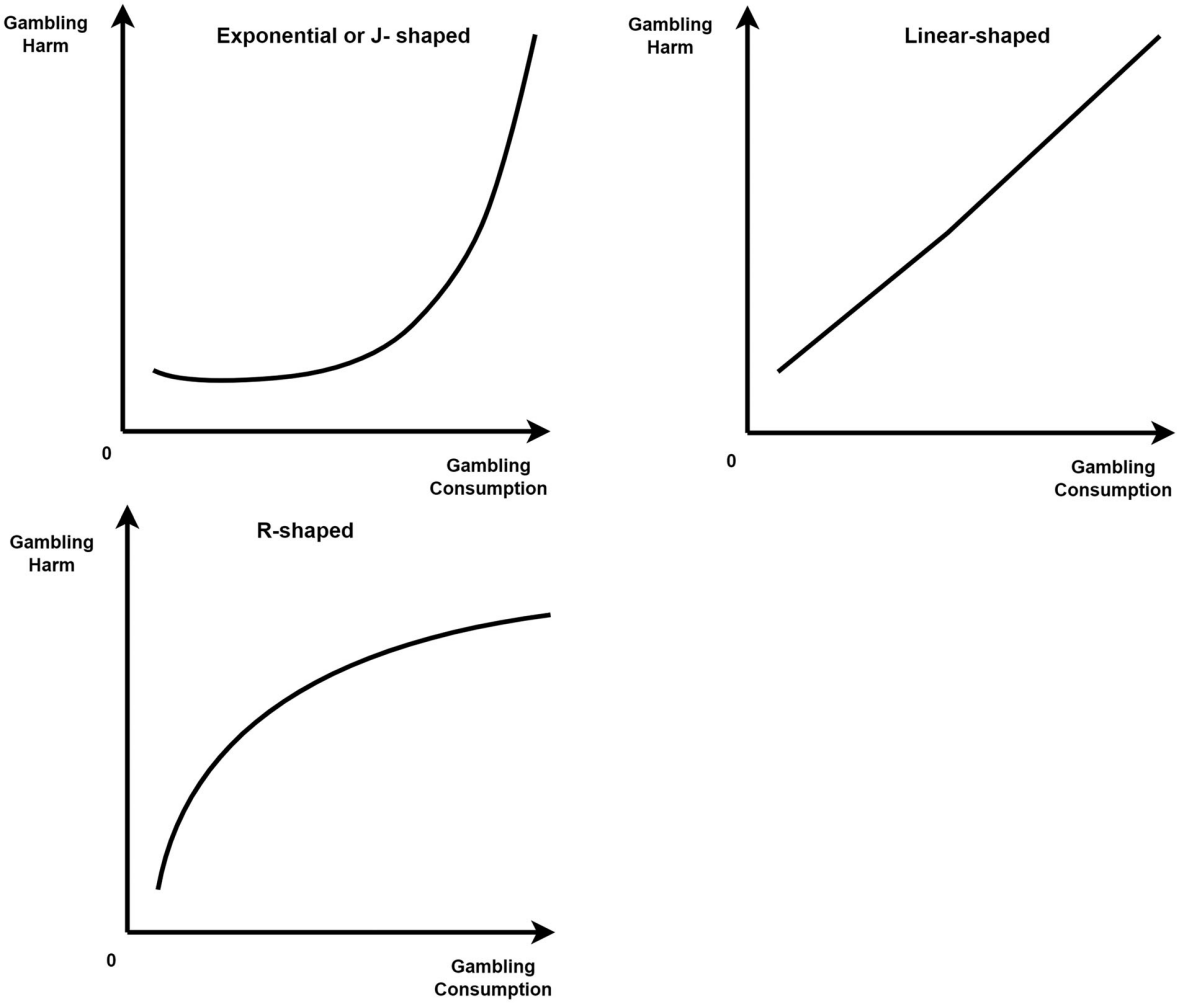
Shape of the risk curves in gambling.

Seven studies were identified that examined the shape of the risk curves between gambling consumption and experience of gambling harms (see Appendix C). Several authors (e.g. Currie et al. 2017; Currie 2019) referred to the risk curves as J-shaped, where risk of harm remains low and stable at low and medium exposure levels and mainly increases quite steeply at higher exposure levels. In other areas of epidemiology, J-shaped risk curves imply that risk of harm is lower at low/moderate level of exposure compared to no or very little exposure (Chokshi et al. 2015), often inferred as a protective effect of low/moderate exposure (as for instance a cardioprotective effect of moderate alcohol consumption, e.g. Grønbæk et al. 2021). In order to be consistent with the broader epidemiological literature, we use the term *exponential* to describe these risk curves. A summary of the potential risk curves observed in our review is provided in Figure 3.

In general, a risk curve is a visualization of a dose-response relationship, plotted as harm risk (on the Y-axis) against a continuous exposure measure (on the X-axis). However, as critiqued by Markham et al. (2016) and Greenwood et al. (2021), several previous studies (e.g. Currie et al. 2006, 2008; Currie 2019) presented ordered-categorical exposure data as though categories were of equal magnitude, and hence the previously identified exponential risk curves were an artifact of the categorical data (Greenwood et al. 2021).

Because of these methodological limitations, as well as theoretical considerations (whereby the TCM requires a continuous consumption measure), we primarily summarize evidence where continuous or re-scaled categorical measures have been used. We mention only in brief other studies and given their methodological approach we advise caution with the interpretation of these in relation to the TCM.

Using continuous or re-scaled categorical measures of gambling consumption, Greenwood et al. (2021) analyzed data from Tasmania, Australia and found evidence of linear, rather than exponential, risk curves. A similar approach was taken by Markham et al. (2016) who analyzed risk of problem gambling employing data from four jurisdictions (Australia, Canada, Finland, and Norway). The authors concluded that risk curves for total gambling losses and for different gambling activities are likely to be linear or r-shaped, and they found that losses on electronic gaming machines (EGMs) were more strongly associated with experiencing harm than other activities such as table games.

Five studies, (Currie et al. 2017; Currie 2019; Finnsdóttir 2020; Louderback et al. 2021; Räsänen et al. 2016) found mostly exponential risk curves, though some of the gambling consumption measures were treated as categorical. Thus, these findings should be interpreted with caution in relation to the TCM given their methodology, as discussed above. For instance, a study by Finnsdóttir (2020) used discrete and categorical measures of gambling consumption. The author finds exponential risk curves for gambling expenditure, frequency, number of gambling activities, and percent of income spent on gambling.

### Concentration of gambling consumption

Six studies (Orford et al. 2013, Tom et al. 2014; Fiedler et al. 2019; Forrest and McHale 2022; Grönroos et al. 2022; Wardle et al. 2022) were identified which examined the extent to which gambling consumption is concentrated among individuals experiencing moderate risk/problem gambling (MR/PG) or is highly concentrated among a few individuals, (that is the ‘vital few’ typically described as small percentage of a player pool responsible for a majority of gambling revenue). All used cross-sectional analyses. Despite MR/PG constituting a small proportion of the total samples, all showed that gambling expenditure was highly concentrated among those experiencing MR/PG (see Appendix D). One study (Grönroos et al. 2022) found that those experiencing problem gambling (2.8%) accounted for 18.8% of gambling expenditures, and that half of all gambling expenditures stemmed from the 4.2% of gamblers with the highest expenditures. Almost half (46%) of those experiencing problem gambling were among the 4.2% with the highest expenditures, and conversely 29% of those with the highest expenditures were those experiencing problem gambling, suggesting a correlation but no complete overlap between high gambling expenditures and problem gambling. A similar finding was reported by Tom et al. (2014). Their finding, based on actual digital account data from players (as opposed to self-report data), suggests that the proportion of revenue attributable to people experiencing gambling problems was over 70% for online casino games.

### Concentration of consumption by gambling type

Five studies examined concentration among different forms of activity (Orford et al. 2013; Tom et al. 2014; Fiedler et al. 2019; Forrest and McHale 2022; Wardle et al. 2022). These studies found that gambling consumption concentration varied by type of gambling activity. The concentration among those experiencing MR/PG appeared to be higher for continuous games (e.g. casino games/slots) than lotteries (see Appendix D).

However, caution is needed in synthesizing results from these studies. First, the concentration measures used in these studies are not comparable. Second, studies differed with regard to measurements and analytical methodologies used. For instance, two studies used Gini coefficients, while another study examined the Pareto principle.^4^ One study examined gambling expenditure attributable to top 1%, top 10%, and top 20% of highest spending players, and the remaining two studies assessed the concentration of gambling activity using the following: cumulative gambling expenditure, and percentage of days play/spending attributable to people experiencing gambling problems (including moderate risk gamblers).

#### The total consumption model

We identified four studies from our search strategy that specifically looked at the application of the basic and extended versions of the TCM (Hansen and Rossow 2010; Markham et al. 2014; 2017; Rossow 2019). Given that all three primary studies were included in Rossow’s (2019) review we only briefly mention the key findings from these studies. Notably, Markham et al. (2014) observed a close association between EGM expenditure and prevalence of problem gambling using Australian cross-sectional data, whereas the second study (Markham et al. 2017) employed data from various calendar years and states in Australia and found a similar relationship between the two. Hansen and Rossow (2010) assessed the impact of prohibition of note acceptors on gambling behavior among Norwegian adolescents. The authors found that a reduction in gambling frequency was associated with a reduction in prevalence of problem gambling, in line with the TCM. Finally, a comprehensive review by Rossow (2019) identified 12 empirical studies that examined the TCM or provided relevant data. All but one of these studies found empirical support for the TCM; that is, a positive association between population gambling consumption and prevalence of excessive or problem gambling.

## Discussion

The current review extends what is known about the nature of the relationship between total consumption and gambling harm by assessing the risk curves in these relationships, concentration of gambling consumption, and the general applicability of the TCM.

### Key findings

Several key findings emerge from this review. First, there is mixed evidence on the shape of the risk curves, with some studies finding a linear or r-shaped risk curve, which implies that a considerable fraction of gambling harm might occur at moderate or intermediate levels of gambling consumption. That said, there are some limitations of the risk curve studies that might be important to note. For example, Markham et al. (2016) assessed the risk curves for gambling losses and used bootstrapping which reduces the influence of outliers. This may lead to an underestimation of the harms and thus alter the shape of the resulting risk curve. This, along with the methodological issues of using categorical data for risk curve analysis, suggests that it is essential to develop a common set of methodological standards to be applied to studies assessing risk curves going forward. This should include agreement on the appropriate treatment, scaling and presentation of categorical data used for these purposes, analytical methods, and treatments of outliers. Another notable gap is the lack of risk curve studies for other specific harms from gambling, including problems with regard to family, mental health, debts, or crime.

Second, six studies found considerable concentration of gambling consumption among MR/PG or among a ‘vital few’ consumers, which is in line with previous studies published before 2010 (See Fiedler et al. 2019 for a review). Five papers examined concentration of consumption by product type and showed that concentration among MR/PG was least for lotteries and largest for continuous forms of gambling, like slot machines or casino games, among others. This corresponds to previous findings of very skewed gambling consumption distributions. For instance, Tom et al. (2014) found for several online gambling forms that 80% of gambling consumption was attributable to 5% of gamblers. Moreover, while gambling consumption was concentrated among MR/PG and among high-consumption gamblers, these two groups overlap only in part (e.g. Tom et al. 2014). This corresponds with findings from risk curve studies, suggesting that gambling harms occur also at moderate levels of gambling consumption.

Relatedly, the modifying influence of gambling formats, venues, and gambling modes, may impact the applicability of the TCM. For instance, for gambling products with systematic high gambling concentration (such as EGMs), the association between total consumption and prevalence of excessive consumption is likely to be higher than for say, lottery. That said, this does not necessarily imply that the TCM is less valid for products where distribution of consumption is less skewed. As long as the distribution follows a fairly regular pattern across populations with different mean (or total) consumption, we expect a positive correlation between mean consumption and prevalence of either excessive consumption or prevalence of harms.

Gambling behaviors, including the experience of harms, are dynamic and change over time, with people moving within the risk spectrum. Several factors may influence the prevalence of problem gambling (including but not limited to the introduction of new gambling products or substantial changes to gambling availability). Whilst examination of the relationship between gambling participation and problem gambling under conditions where gambling availability has increased has been undertaken (Abbott et al. 2014, 2018), the extent to which these factors also impact the relationship between total consumption (rather than participation prevalence) and harm from gambling is still underexplored.

Although still meager, the gambling literature on TCM and related issues seemingly resembles, in several respects, the alcohol literature. Specifically, the distribution of consumption is skewed, yet harms occur not only at high – but also at moderate consumption levels. Moreover, at the population level, consumption level is correlated with level of harm; the higher the total consumption, the higher the number of excessive consumers and the harm level (Rossow and Mäkelä 2021). Equally, there are some situations in both alcohol studies and gambling where the TCM may be less aplicable. For alcohol consumption, a policy of strict individual rationing, as was the case in Sweden 1919 – 1955 (Norström 1987), impacts markedly on the tail of the distribution and the extent of exessive consumption. In this situation, total consumption may vary considerably, whereas excessive consumption and related harms cannot vary to the same extent. Correspondingly, it seems likely that strict and mandatory individual limitations on winnings and losses on gambling would strongly affect the tail of the distribution, but not necessarily total consumption as much.

That said, it is also important to elaborate areas where the application of the TCM to gambling may need more specific consideration. For gambling, harm risk seems to be higher for some products (e.g. continuous games such as electronic gambling machines [EGMs] than for others; see Markham et al. 2014) raising important questions about whether the empirical support for TCM and its implications for gambling policy are stronger for some forms of gambling.

#### Practical and theoretical implications

This review has several main implications. First, with regard to prevention of gambling harms, there is nascent literature in support of the validity of the TCM for gambling, implying that gambling harms could be prevented by employing measures that effectively reduce total consumption of harmful gambling products. Particularly for gambling products where the risk curve is linear or r-shaped, population targeted measures may be effective to curb the overall harm level. Such measures, when effective, will likely impact gamblers at all levels of consumption.

From the alcohol policy literature, there is good evidence that universal control policy measures, including regulation of price and physical availability, not only impact the large majority of moderate consumers but also reduce consumption and harm among the heavy consumers (Babor et al. 2023). Although sparse, there is some evidence that universal control policy measures for gambling, may impact consumers at all levels of consumption. In Norway, in the early 2000s, EGMs were easily available, particularly among young people, accounting for a large fraction of total gambling consumption (Rossow and Hansen 2016). Following a ban on note acceptors on EGMs in 2006, mean total gambling frequency among young people decreased significantly, as did gambling frequency at low, intermediate, and high levels (Hansen and Rossow 2012). However, considering the skewed distribution of consumption for some products, it may be appropriate to implement various prevention strategies focusing on high-risk groups in concert with universal measures.

Overall, this is congruent with the public health approach presented in Korn and Shaffer (1999) and Delfabbro and King (2020) suggesting that interventions should focus on the whole continuum, especially because there are more low-or -moderate risk individuals than high-risk and thus the overall number of individuals experiencing harm might be much higher among low- or -moderate risk than high-risk individuals. This is also imperative from a prevention viewpoint to prevent these low- or -moderate risk individuals from becoming more serious high-risk gamblers (Delfabbro and King 2020).

A second implication is with regard to our understanding of problem gambling. Evidence of a close association between total gambling consumption and the number of people experiencing MR/PG suggests that experience of harms is not just a result of individual vulnerability, but also that of overall gambling activity in society. Thus, it may be argued that the society at large and governments also carry a responsibility for preventing gambling harms by limiting total gambling consumption (see Blaszczynski et al. 2011). The converse discourse, often put forward by the gambling industry, suggests that the onus is on individuals to gamble responsibly (Livingstone and Rintoul 2020). Such arguments shift the responsibility from the gambling industry to the individual gambler. While many governments now recognize the role of the gambling industry in causing gambling harm, they have made no major attempts to curtail the supply or the demand of gambling products (Marionneau et al. 2022).

#### Directions for future Research

Our review has several recommendations for further research. In all three areas covered here, there is a clear need for more studies to strengthen the evidence base. Well-designed risk-curve studies, that also examine risks by various product types, demographic groups, and risks for various types of gambling harms are needed. More knowledge is also required about the population distribution curves for gambling behavior, both with regard to various measures for assessing concentration, and with regard to various gambling products. Empirical studies examining the validity of the TCM for gambling in its basic and extended version are also warranted. A set of methodological standards for conducting high-quality analyses on the TCM are needed, including recognition that measuring gambling consumption is not the same as measuring gambling participation prevalence – the latter looks at how many people are gambling over a given time period, it does not measure how much gambling they are doing either individually or collectively. Better and more consistent measurement of total gambling consumption is needed to fully explore the application of the TCM to gambling. Likewise, as we increasingly recognize the broader range of harms associated with gambling, the TCM for gambling could be extended to assess the relationship between total consumption and wider gambling harms, though the latter also requires more systematic and rigorous measurement.

Better understanding of the relationship between gambling consumption and its associated harm could be developed through investments into generating more data (cross-sectional and longitudinal) and analyses of that data, particularly in locations where this has been under-researched, such as in low- and -middle income countries. This would allow us to assess epidemiological changes over time and across populations, allowing us to monitor these relationships more closely.

#### Limitations

One limitation when attempting to assess the applicability of the TCM to gambling is that the epidemiological evidence during times of gambling expansion and its relationship to harms is varied. For example, adaptation theory has been used to examine the relationship between changing gambling supply and harms, postulating that as new forms of gambling activities develop, gambling harm will increase in the short run, however as people adapt over time these harms begin to decline (LaPlante and Shaffer 2007; Sulkunen et al. 2019). This theory and evidence runs counter to the hypotheses postulated by the TCM. However, the evidence base examining how the relationship between gambling harms changes in relation to increased supply and availability of gambling is hampered by poor and inconsistent measurement of consumption, tending to rely on point-based prevalence estimates of gambling participation as a proxy measure for gambling consumption (Sturgis and Kuha 2021). Such measurement error bias highlights an important problem that researchers face when trying to test the hypothesis of the TCM.

There are several further limitations of this review. The review is based on a relatively small evidence base of high-income countries; thus, the findings of this review may not be generalizable to other regions in the world such as low- or -middle income countries. Moreover, the majority of the datasets analyzed by the included studies are more than two decades old and thus might not be considered relevant for today, especially given the significant increase in online gambling activity. Another important limitation is that we only considered English language papers, thus we may have missed some relevant non-English publications.

## Conclusion

As gambling opportunities expand worldwide, policy and academic attention should focus on the impact of this upon gambling harms. Rossow (2019) previously highlighted the small, but consistent body of evidence supporting the application of the TCM to gambling. Our review extends and updates this work, finding further evidence for some of the central tenets which underlie this theory. A small and consistent evidence base continues to show that gambling is highly concentrated among a few individuals, especially those experiencing MR/PG, and that this is particularly evident for certain activities. Evidence on the shape of risk curves both for gambling overall and for specific activities needs further investigation and would benefit from collaborative effort to devise a common set of methodological standards to guide this analysis. Whilst evidence on the TCM model and its assumptions is still sparse, what evidence exists suggests that the TCM may better apply for some activities than others. The activities where the TCM has greatest potential application are those which are continuous forms of gambling with high degrees of concentration of consumption, where the shape of the risk curve (r-shaped, linear) would suggest that a population-based approach to harm minimization is warranted. However, such a change in gambling harm prevention strategy (shifting the focus from individuals alone to include population-level consumption of gambling) will entail a huge cultural shift as many people (governments included) still believe that it is the responsibility of the individual to reduce their gambling activity, disregarding the role of the gambling industry. That said, the current evidence base substantiating the applicability of the TCM in gambling is nascent and thus more research is necessary in order to explore the best strategies to prevent population-level of gambling harms.

## Supplementary Material

Supplemental Appendix

## Footnotes

1 Data from Global Betting and Gaming Consultants (GBGC), owned by Regulus.

2 Note: Betting trajectory is the estimated slope coefficient of betting frequency on the amount wagered. Daily betting variability is the standard deviation of betting frequency on the amount wagered. Net outcome of gambling is the difference between total amount wagered and total winnings. Number of different types of gambling refers to ten gambling forms: Lotto, EGMs, Scratch tickets, Betting, Betting on own performance, Lottery, Poker, Other card games, Bingo, and Casino gambling. Continuous gambling types are skill-based gambling forms which have a high event frequency (e.g., EGMs). Number of gambling activities is the number of gambling types participated in (e.g., in the last month). Percent of income spent on gambling is the proportion of income spent on gambling. Frequency of gambling refers to the number of days (e.g., per month) individuals participates in gambling. Gambling expenditure is the total (e.g., weekly, monthly) expenditure on gambling.

3 Note: Problem Gambling Severity Index (PGSI), South Oaks Gambling Screen (SOGS), NORC or related DSM Screen for Gambling Problems including the Brief Biosocial Gambling Screen (NORC or related DSM Screen measures), Canadian Problem Gambling Index (CPGI), Lie/Bet Questionnaire (Lie/Bet), Problem and Pathological Gambling Measure (PPGM), Short Gambling Harm Screen (SGHS), and concentration of expenditure- top 1%, top 10%, and top 20%.

4 Based on Pareto (1896). Cours d'économie politique: professé à l'Universi̧té de Lausanne (Vol. 1 and 2). F. Rouge.
