# Supplementary material for: Gambling consumption and harm: a systematic review of the evidence
Source: Addict Res Theory. 2023 Aug 2;32(3):194–203. doi: 10.1080/16066359.2023.2238608 (PMC11104743; doi:10.1080/16066359.2023.2238608)
Supplement: Supplemental Appendix [file IART_A_2238608_SM3506.docx]

# **Appendices**

**Search strategy**

Appendix A Search strategy for the TCM

| **Research questions** | What evidence is there about the current shape of the risk curves in gambling? What do we know about the current shape of gambling consumption and its concentration? What does recent evidence tell us about the application of total consumption models to gambling? |
| --- | --- |
| **Methods** | |
| **Search databases:** | |
| Academic | Medline; PsycINFO; Web of Science |
| Grey literature | Google Scholar; GoGamble Aware InfoHub; Gambling Commission; GambLib (Gambling Research Library); World Health Organisation (WHO); Public Health England (PHE); Public Health Scotland (PHS); Public Health Wales (PHW); Public Health Agency (PHA); Victoria Responsible Gambling Federation; Ministry of Health New Zealand; Gambling Research Exchange Ontario; International Center for Responsible Gaming; European Gaming and Betting Association. |
| **Search terms (full text)**  **Risk curves** | 1. “risk-curve*” OR “J-shape*” OR “linear*” OR “exponential*” OR “r-shape*” OR “curvilinear” OR “accelerating” 2. “Gambl* loss*” OR “Problem gambl*” OR “Pathological gambl*” OR “compulsive gambl*” OR “disordered gambl*” OR “intemperate gambl*” OR “Gambl* prevalence” OR “Excessive gambl*” OR “Gambl* frequency” OR “Gambl* activity” OR “Mean gambl* expenditure” OR “Problem Gambling Severity Index” OR “Gambling Symptom Assessment Scale” OR “Problem and Pathological Gambling Measure” OR “NORC DSM Screen for Gambling Problems” OR “gambling disorder” OR “Problem and Pathological Gambling Measure” OR “South Oaks Gambling Screen” OR “Canadian Problem Gambling Index” OR “Problem and Pathological Gambling Measure” OR “Victorian Gambling Screen” NOT “linear regression” NOT “linear model*” NOT “linear random” NOT “linear mixed” 3. 1 AND 2   Limitations:  From 2010 onwards  English language papers |
| **Search terms (full text)**  **Concentration** | - - - 1. “Concentration of revenue” OR “Revenue” OR “GINI index”       2. “Gambl* loss*” OR “Problem gambl*” OR “Pathological gambl*” OR “compulsive gambl*” OR “disordered gambl*” OR “intemperate gambl*” OR “Gambl* prevalence” OR “Excessive gambl*” OR “Gambl* frequency” OR “Mean gambl* expenditure” OR “Problem Gambling Severity Index” OR “Gambling Symptom Assessment Scale” OR “Problem and Pathological Gambling Measure” OR “NORC DSM Screen for Gambling Problems” OR “gambling disorder” OR “Problem and Pathological Gambling Measure” OR “South Oaks Gambling Screen” OR “Internet Gaming Disorder Scale” OR “internet gaming disorder” OR “Canadian Problem Gambling Index” OR “Problem and Pathological Gambling Measure” OR “Victorian Gambling Screen”       3. 1 AND 2   Limitations:  From 2010 onwards  English language papers |
| **Search terms (full text)**  **Total consumption model** | 1. gambling OR betting OR wager* 2. total consumption OR single distribution 3. (“Problem gambl*” OR “Pathological gambl*” OR “compulsive gambl*” OR “disordered gambl*” OR “intemperate gambl*” OR “Gambl* prevalence” OR “Excessive gambl*” OR “Gambl* frequency” OR “Mean gambl* expenditure” OR “Problem Gambling Severity Index” OR “Gambling Symptom Assessment Scale” OR “Problem and Pathological Gambling Measure” OR “NORC DSM Screen for Gambling Problems” OR “gambling disorder” OR “Problem and Pathological Gambling Measure” OR “South Oaks Gambling Screen” OR “Internet Gaming Disorder Scale” OR “internet gaming disorder” OR “Canadian Problem Gambling Index” OR “Problem and Pathological Gambling Measure” OR “Victorian Gambling Screen” OR “gambling harm*”) NOT “Cambridge gambling task” NOT “IOWA gambling task” 4. 1 AND 2 AND 3   Limitations:  From 2010 onwards  English language papers |
| **Inclusion criteria:** | |
| **Types of studies** | Reviews, commentaries, and empirical studies published from 2010 onwards (to coincide with broader development of online gambling)  Primary or secondary data analyses on the TCM in gambling.  English language papers.  Studies that either reported TCM or TCM using either risk curves, or concentration measures. |
| **Types of participants** | children, adolescents, and adults |
| **Types of articles** | Reviews and empirical studies that present evidence on the relationship between population mean gambling and prevalence of excessive/problem gambling.  Commentaries/discussion articles. |
| **Types of comparisons** | Gambling modalities (online versus land-based)  Gambling activities |
| **Types of outcome measure** | Population gambling mean (including measures such as rates of past year gambling; past gambling participation; gambling frequency; gambling expenditure; and gambling losses);  Prevalence of excessive gambling (proportion above a fixed cut-off on a continuous gambling indicator) or problem gambling measures (self-reported harms based on the PGSI measure; an extended version of the TCM; Rossow, 2019);  Prevalence of excessive gambling |
| **Other** | English language  No country restrictions |
| **Data collection and analysis:** | |
| **Selection of studies** | This review was conducted and reported according to PRISMA (Preferred Reporting Items for Systematic Reviews and Meta-Analyses) guidelines (Moher et al., 2009). |
| **Data extraction** | Data extracted for each article included:  Author/Title/Year of publication  Country/region of study  Objectives  Dataset/Period covered/Sample size  Population  Game genre  Analyses type  Measure population mean gambling  Measure excessive gambling  Methodology  Findings  Limitations |
| **Assessment of methodological quality** | The study methodology was assessed informally based on the data and the methods that were used. |
| **Data synthesis** | The characteristics of the included studies were summarised in a table and using charts. |

Appendix B PRISMA diagram

Records after duplicates removed

N=553

**Eligibility**

**Included**

Full-text articles assessed for eligibility

N=21

Studies included in review

N=10

Additional records identified through cross-referencing

N=7

**Identification**

**Screening**

Records identified through database searching

N=920

Studies included in the analysis of the review

N=17

k

Records excluded by abstract
N=532

Records excluded by full-text

N=11

Definition of problem gambling, N=2

Tests other theories, N=4

Measures of gambling consumption, N=1

Does not test the TCM assumption, N=4

**Note: RC: risk curves; CC: concentration literature; and TCM: Total Consumption model**

Appendix C Key characteristics of the included studies

| Author | Country | Study design | Population | Sample size | Measures of gambling consumption | Measures of gambling  harm | Analysis type | Findings |
| --- | --- | --- | --- | --- | --- | --- | --- | --- |
| Risk curve literature | | | | | | | | |
| Currie et al 2017 | Canada | Longitudinal | Adolescents  /Adults | Quinte Longitudinal Study (n=3,054)  Lifestyle and Lifecycle Project (n=809) | Frequency; expenditure;  % of income spent  (Monthly) | CPGI  (2 or more harms) | Risk curves presenting categorical gambling consumption and % reporting two or more harms | Exponential risk curves for both datasets |
| Currie et al 2019 | Canada | Cross-sectional | Adults | Gambling prevalence surveys pooled from individual studies N=7,675 | Expenditure (Monthly) | Gambling harms derived from  PGSI  (Bet more than can afford; health; finance; gambling problem) | Risk curves presenting categorical gambling consumption and % reporting gambling related harms, namely bet more than could afford, health, finance, and gambling problems | Exponential risk curve |
| Greenwood et al 2021 | Tasmania, Australia | Cross-sectional | Adults | The fourth Social and Economic Impact Study of Gambling in Tasmania N=2873 gamblers | Frequency; expenditure; % of income spent  (In the last 12 months) | PGSI  SGHS  (Continuous and binary harm measures, 2 or more harms) | Risk curves presenting continuous (standardised) & categorical gambling consumption (re-scaled to the mid-point)  and gambling harms measures (standardised with continuous gambling consumption measures)  Linear (with continuous outcomes) and logistic (with binary outcomes) regression analyses, all were bootstrapped | Exponential risk curve when gambling consumption enters as a categorical variable.  When gambling consumption is  re-scaled to the mid-points or when it enters as a continuous variable, risk-curves no longer appeared to have an exponential-shape.  The relationships were either positive linear or negative quadratic. |
| Finnsdóttir, 2020 | Iceland | Cross-sectional | Adults  [18-70] | N=2,028 | Frequency (days per month); expenditure per month; % of income spent; number of gambling activities; and number of continuous gambling types | PGSI  (Includes five types of harms: i) Emotional /  psychological, ii) Financial, iii) Physical, iv) Relational, and v) 2 or more harms) | Risk curves presenting continuous and categorical gambling consumption and % of gambling harm measures | Exponential (gambling expenditure, frequency, number of gambling activities, % of income spent) and linear-shaped (number of continuous gambling types) risk curves |
| Louderback et al 2021 | Online customers on bwin Interactive Entertainment (based on three secondary datasets) | Longitudinal | Adults | Between 1,772 and 44,754 | Frequency;  amount wagered; net outcome of gambling  (Monthly);  % yearly household income wagered online; betting escalation (during a player’s first six months of activity); betting variability  (in one day) | BBGS positive  (Derived from the DSM-IV  criteria for Gambling Disorder) | Risk curves presenting gambling consumption (categorical) and % screening BBGS positive | Exponential risk curve |
| Markham et al 2016 | Australia (1999), Canada (2000), Finland (2011) and Norway (2002) | Cross-sectional | Adults  [15–74 years] | 10,632 Australians, 3120 Canadians, 4484 Finnish and 5235 Norwegians | Losses  (Monthly) | SOGS  PGSI  NORC DSM  Screen for Gambling Problems | Risk curves presenting continuous gambling consumption and gambling harm (standardised) measures,  Multiple linear regression adjusted for key covariates, and mixed-effects linear models; all analyses were bootstrapped | r-shaped in Australia, Canada, and Finland; linear in Norway. Gambling losses and its corresponding harm were strongly correlated for EGM activity. |
| Räsänen 2016 | Finland | Cross-sectional | Adolescents  (12–17-year-olds) | 1,827 | Frequency; number of different type of gambling activities  (In the last 12 months); expenditure (Weekly);  largest expenditure on gambling activities  (In one day) | SOGS Gambling Screen revised for adolescents (Four or more harms were considered as people experiencing gambling-related problems) | Risk curves presenting categorical gambling consumption and % experiencing 4 or more gambling harm measures | Exponential risk curve |
| Gambling concentration literature | | | | | | | | |
| Fiedler et al 2019 | Finland | Cross-sectional (3 countries) | Adults (18 years and older) | 8,794 (French); 7,529 (Quebeckers); 2,923 (Germans) | Expenditure | PGSI  DSM-IV  (Problematic gamblers: PGSI 3-7 or DSM 3-4; Pathological gamblers: PGSI >7 or DSM >4) | Gini coefficients (based on spending) for people experiencing gambling problems and those with symptoms of pathological gambling | A strong positive relationship between GINI coefficient and the share of revenue derived from people experiencing gambling-related problems. |
| Forrest and McHale, 2022 | England, Scotland, and Wales | Cross-sectional (gambling activity) | Adults (18 years and older) | 139,152 | Expenditure | Concentration of expenditure | Top 1%, 10%, and 20% | Expenditure on gambling was highly concentrated. |
| Grönroos et al 2021 | Finland | Cross-sectional | Adults (18 years and older) | 5, 805 | Expenditure | Problem and Pathological Gambling  Measure (PPGM)  (Using PPGM the following were created: i) problem gambling;  ii) at-risk gambling; and  iii) recreational gambling) | Cumulative GE by PPGM  Multinomial logistic regression (Highest expenditure versus  lowest expenditure as the outcome) | GE is highly concentrated. The majority of the revenue comes from at-risk and problem gambling. |
| Orford et al 2013 | England, Scotland, and Wales | Cross-sectional | Adolescents/ adults aged 16 or over | 7,756 | Frequency; expenditure | PGSI  DSM-IV  (PGSI: three or more a ‘moderate risk’ gambler; DSM-IV: three or more of the criteria to be classed as people experiencing gambling problems) | Percentage of days play/ spending attributable to people experiencing gambling problems (and moderate risk) gamblers | People experiencing gambling-related problems tend to play more frequently and have greater losses from gambling. |
| Tom et al 2014 | Internet gambling subscribers | Cross-sectional (sub-samples for Online Casino Turnover, Online Casino Hold, and Online Casino Bets) | Adults | 1,384 | Internet gambling expenditure | BBGS positive  (Derived from the DSM-IV  criteria for Gambling Disorder, includes withdrawal, lying, and borrowing money) | 80/20 division for turnover, hold, and bets | People experiencing gambling-related problems spend a disproportionately higher amount on gambling than those who don’t experience gambling-related problems. |
| Wardle et al 2022 | UK | Longitudinal | Adults | 3,865 (W1), and 2,980 (W2) | Expenditure | PGSI  (Scoring 3 or more) | Gini coefficients (based on spending) for moderate risk and people experiencing gambling problems | People experiencing gambling-related problems spend disproportionately more than those who don’t experience gambling-related problems, with online casino having the greatest level of concentration of expenditure. |
| TCM literature | | | | | | | | |
| Markham et al 2014 | Australia | Cross-sectional (62 EGM venues) | Adults (18 years and older) | 7049 | EGM expenditure | PGSI  (2 of the nine questions) | Binomial rate regression | Almost linear type relationship between EGM expenditure and gambling related harm |
| Markham et al 2017 | Australia | Cross-sectional (Pooled estimates from 41 studies) | Adults (18 years and older) | Between 1253 and 9408 | EGM and casino gambling losses | PGSI  SOGS  (Problem gambling defined as PGSI ≥ 8 or SOGS ≥ 5; moderate-risk problem gambling,  PGSI 3-7 or SOGS 3-4) | Bayesian meta-regression analysis based on 41 cross-sectional studies | Positive adjusted association between EGM and casino gambling losses and problem gambling prevalence |
| Rossow, 2019 | Canada, UK, Norway, US, Australia, New Zealand, and Finland | Review (includes studies that use both cross-sectional and longitudinal datasets) | Adolescents/ adults | n/a | Population mean gambling consumption measures include gambling expenditure, gambling expenditure as a % of household income; EGM expenditure, gambling losses, gambling losses as a % of income, and gambling frequency | Excessive gambling consumption (i.e., proportion above a fixed cut-off on the same continuous gambling measure)  PGSI  SOGS | Review of studies that empirically assessed the relationship between gambling consumption and gambling harm measures | Close association between population gambling mean and gambling harm measures. |
| Hansen and Rossow, 2010 | Norway | Cross-sectional  (3 samples) | Adolescents  (13-19) | 20,000 | Frequency; EGM expenditures | SOGS-RA 4þ LieBet 2 | Multivariate linear regression  Logistic regression models | Following the prohibition of cash acceptors there was a decrease in both gambling frequency and EGM expenditure, as well as problem gambling. |

Appendix D Overview of studies describing concentration of gambling consumption in people experiencing gambling-related problems

| **Activity** | **France (Fiedler et al 2019)** | | | **Quebec (Fiedler et al 2019)** | | | | **Great Britain (Orford et al 2013)** | | | | **Bwin analysis (Tom et al 2014)** | | | |
| --- | --- | --- | --- | --- | --- | --- | --- | --- | --- | --- | --- | --- | --- | --- | --- |
|  | PG/MR prevalence | Proportion of spend attributable to PG/MR | Excess spending (percentage point difference) | | PG/MR prevalence | Proportion of spend attributable to PG/MR | Ratio of proportion of gambling consumption/prevalence of PG/MR | | PG/MR prevalence | Proportion of spend attributable to PG/MR | Ratio of proportion of gambling consumption/prevalence of PG/MR | | Brief bio-social positive (BBGS+) prevalence | Proportion of turnover attributable to BBGS+ | Ratio of proportion of gambling consumption/prevalence of BBGS+) |
| Lotteries | 4.70% | 24.20% | +19.5 | | 2.70% | 10.50% | +7.8 | | 3.30% | 12.90% | +9.6 | | n/a | n/a | n/a |
| Scratchcards | 5.30% | 26.10% | +20.8 | | n/a | n/a | n/a | | 5.90% | 14.50% | +8.6 | | n/a | n/a | n/a |
| Slots/Electronic gaming machines | 9.90% | 41.00% | +31.1 | | 8.70% | 76.30% | +67.6 | | 10.10% | 27.10% | +17.- | | n/a | n/a | n/a |
| Horse racing | 12.10% | 40.20% | +28.1 | | n/a | n/a | n/a | | 6.30% | 22.50% | +16.2 | | n/a | n/a | n/a |
| Poker | 18.60% | 63.30% | +44.7 | | 8.00% | 43.60% | +35.6 | | 20.00% | 30.30% | +10.3 | | n/a | n/a | n/a |
| Casino table games | 15.90% | 76.10% | +60.2 | | 8.30% | 44.10% | +35.8 | | 16.00% | 42.30% | +26.3 | | n/a | n/a | n/a |
| Sports betting | 19.20% | 58.50% | +39.3 | | 8.00% | 16.00% | +8.0 | | 12.80% | 26.60% | +13.8 | | 28.10% | 51.80% | +30.0 |
| Online casino games | n/a | n/a | n/a | | n/a | n/a | n/a | | 21.00% | 30.50% | +9.5 | | 36.50% | 72.10% | +35.6 |
